# The Prognostic Role of IL-6 and RBP4 in Colorectal Cancer

**DOI:** 10.3390/biomedicines13092257

**Published:** 2025-09-13

**Authors:** Małgorzata Żulicka, Daria Piątkowska, Dariusz Grzanka, Klaudia Bonowicz, Dominika Jerka, Maciej Gagat, Paulina Antosik

**Affiliations:** 1 Department of Clinical Pathomorphology, Faculty of Medicine, Collegium Medicum in Bydgoszcz, Nicolaus Copernicus University in Torun, 85-094 Bydgoszcz, Poland; malgorzatazulicka@gmail.com (M.Ż.); dariapiatkowska0@gmail.com (D.P.); d_grzanka@cm.umk.pl (D.G.); 2 Department of Histology and Embryology, Collegium Medicum in Bydgoszcz, Nicolaus Copernicus University in Torun, 85-092 Bydgoszcz, Poland; klaudia.bonowicz@cm.umk.pl (K.B.); dominika.jerka@cm.umk.pl (D.J.); mgagat@cm.umk.pl (M.G.); 3 Department of Morphological and Physiological Sciences, Faculty of Medicine, Collegium Medicum, Mazovian Academy in Płock, 09-402 Płock, Poland

**Keywords:** Il-6, RBP4, colorectal cancer, prognostic factor

## Abstract

**Background/Objectives:** Colorectal cancer (CRC) is one of the leading causes of cancer-related mortality worldwide. Inflammation and metabolic dysregulation, particularly those related to obesity, have emerged as critical contributors to CRC progression. Interleukin-6 (IL-6) and retinol-binding protein 4 (RBP4), an adipokine involved in metabolic regulation, may be key mediators of these processes. This study aimed to evaluate the expression levels of IL-6 and RBP4 in CRC tissues and their associations with clinicopathological features and overall survival. Furthermore, in silico analyses were performed to explore the molecular networks and signaling pathways related to both biomarkers. **Methods:** Immunohistochemical staining of IL-6 and RBP4 was conducted in 118 CRC and matched adjacent normal tissues. Expression levels were assessed using the H-score system and correlated with clinical parameters. Survival analysis was performed using Kaplan–Meier curves. In silico analyses were based on RNA-seq data from TCGA and included pathway enrichment, gene co-expression, and protein–protein interaction networks. **Results:** IL-6 and RBP4 expression were significantly elevated in tumor tissue compared to adjacent normal mucosa. High IL-6 expression correlated with age and obesity measures, while RBP4 expression showed significant associations with pT stage, lymph node involvement, TNM stage, and obesity-related parameters. Kaplan–Meier analyses indicated shorter overall survival in patients with high IL-6 or RBP4 expression. In silico analysis confirmed upregulation of IL6 and RBP4 in CRC and highlighted immune-related pathways for IL-6 and developmental signaling for RBP4. **Conclusions:** Elevated expression of IL-6 and RBP4 in CRC tissue is associated with adverse clinical features and reduced survival, underscoring their potential role as prognostic biomarkers. These findings support the involvement of inflammation and metabolic dysfunction in CRC progression and suggest IL-6 and RBP4 as candidates for future targeted therapeutic approaches.

## 1. Introduction

Colorectal cancer (CRC) is one of the most common cancers worldwide, ranking fourth in incidence and fifth in mortality. In 2022, 1,142,286 new CRC cases were diagnosed globally, and projections estimate that by 2040, this number will rise to 1,916,781 [1,2]. A major challenge in CRC management is the often-late onset of clinical symptoms, which hampers early diagnosis and reduces the effectiveness of treatment strategies [3,4].

Chronic inflammation has emerged as a key driver in CRC development and progression. Immune cells infiltrating the tumor microenvironment (TME) release numerous pro-inflammatory cytokines, including interleukin-6 (IL-6), which amplify local inflammation [5]. IL-6, produced by immune cells (mainly macrophages), tumor cells, and intestinal epithelial cells, orchestrates immune responses, regulates lymphocyte maturation, and contributes to macrophage differentiation [6,7]. In the CRC microenvironment, IL-6 stimulates cancer-associated fibroblasts (CAFs), which in turn further secrete IL-6, creating a positive feedback loop that sustains chronic inflammation [5,6]. Moreover, IL-6 promotes angiogenesis, enhances cancer cell motility through hepatocyte growth factor (HGF) signaling, and impairs anti-tumor immunity by generating dysfunctional natural killer (NK) cells [8,9,10,11,12,13]. Elevated IL-6 levels have been associated with larger tumor size, advanced tumor stage, metastasis, poor prognosis, and increased recurrence risk in esophageal cancer, multiple myeloma, and renal cell carcinoma, among others [7,11,14].

Interestingly, growing evidence points to the role of metabolic dysregulation and adipokines in modulating the inflammatory TME and influencing cancer progression. One such molecule is retinol-binding protein 4 (RBP4), a serum transporter of retinol secreted primarily by the liver and adipocytes [15,16,17]. Recent studies have highlighted that RBP4 is not only a metabolic regulator but also a contributor to tumor biology. Mechanistically, RBP4 can activate macrophages via exosomal signaling, trigger STRA6-mediated Janus kinase (JAK)/STAT3/STAT5 activation, and promote pro-inflammatory responses that support tumor progression, migration, and metastasis [17,18,19]. In gastric cancer cells, RBP4 expression is upregulated, and its silencing inhibits tumor proliferation, migration, and invasion [20]. RBP-4 also increases insulin resistance by reducing phosphatidylinositol 3-phosphate kinase, leading to the formation of pancreatic adenocarcinoma [21]. In CRC, elevated RBP4 expression in tissue correlates with metastasis and unfavorable prognosis [15,16,22]. Furthermore, RBP4-driven endothelial activation enhances vascular permeability and angiogenesis, contributing to tumor growth and dissemination [19]. Notably, RBP4 links adiposity and insulin resistance to cancer biology, with its role being more pronounced in individuals with lower BMI due to adipose tissue dysfunction in obesity [19,21,23,24,25].

In this study, we aimed to investigate the expression patterns and potential clinical significance of IL-6 and RBP4 in colorectal cancer. The initial phase involved the immunohistochemical analysis of IL-6 and RBP4 expression in CRC tissues and adjacent non-cancerous tissues. Protein expression levels were evaluated based on our own immunohistochemical data, while associations with clinicopathological parameters and overall survival (OS) were systematically analyzed. To broaden our understanding, bioinformatic analyses were conducted using publicly available transcriptomic datasets to assess IL-6- and RBP4-related transcriptional landscapes and their potential regulatory networks. Functional enrichment and pathway analyses were performed to identify biological processes, molecular functions, and cellular components associated with IL-6 and RBP4 expression. By integrating experimental and bioinformatic approaches, this study aims to define the prognostic value of IL-6 and RBP4 expression in CRC and to provide novel insights into their roles in colorectal tumorigenesis and potential as therapeutic targets.

## 2. Materials and Methods

### 2.1. Patients and Tissue Material

This study was conducted on tumor and adjacent non-tumor tissue samples collected from patients undergoing colectomy in 2015–2021 at the Department of Surgery of Dr. J. Biziel from the University Hospital No. 2 in Bydgoszcz. Patients diagnosed with colorectal adenocarcinoma were included in this investigation, while the exclusion criteria were as follows: hereditary colorectal cancer, neoadjuvant chemotherapy before resection, and reoperation due to recurrent cancer. The study group included 118 patients (47 females, 71 males) with an average age of 68 years at operation (median 68, range 27–89). Herein, pathologic TNM stage is based on the American Joint Committee on Cancer (AJCC) 8th edition. Histologically normal tissues adjacent to the tumor were available for 42 cases, and these constituted the control group. Clinical follow-up data, including patient survival status, were collected from hospital records. OS was defined as the time from surgery until the last recorded follow-up or death. The end date for survival analysis was set at 25 March 2025.

This study was carried out in accordance with the guidelines of the Declaration of Helsinki. The protocol was approved by the Nicolaus Copernicus University Ethics Committee (no. 454/2021; date of approval: 14/09/2021).

### 2.2. Tissue Microarrays and Immunohistochemical Staining

Immunohistochemical (IHC) staining was performed on tissue microarrays according to the previously described protocol by Antosik et al. IL-6 and RBP4 proteins were stained in BenchMark^®^ ULTRA (Roche Diagnostics/Ventana Medical Systems, Tucson, AZ, USA) using ultraView Universal DAB Detection Kit (Roche Diagnostics/Ventana, Tucson, AZ, USA) according to the previously validated protocol [26]. Tissue sections were incubated with primary rabbit polyclonal anti-IL-6 antibody (dilution 1:100, cat. no: ab6672, Abcam, Cambridge, UK) and rabbit polyclonal anti-RBP4 antibody (dilution 1:250, cat. no: 5950-RBM23-P1ABX, Thermo Fisher Scientific, Waltham, MA, USA). For IL-6, positive control staining was performed on sections of human placenta, with additional validation confirmed by the presence of internal control staining in macrophages. For RBP4, human liver tissue served as the positive control. Negative controls were obtained by omitting the primary antibody. All antibody validations were carried out in accordance with the manufacturer’s recommendations.

### 2.3. Evaluation of Immunohistochemical Staining

The protein expression was assessed at 20× objective magnification in a blinded manner by two independent researchers, including a senior pathologist (DG) (inter-observer agreement: IL-6, κ = 0.760; RBP4, κ = 0.781), using the DP600 scanner (Roche Diagnostics/Ventana Medical Systems, Tucson, AZ, USA). Immunohistochemical expression was evaluated using the H-score system, which combines staining intensity (scored 0–3) and the percentage of positively stained cells. The final score ranged from 0 to 300. For analytical purposes, protein expression was dichotomized into low (negative) and high (positive) categories based on discriminatory cut-off values determined using the Evaluate Cutpoints software (R version 4.4.2) [27]. The cut-off values were set at <225 for low and ≥225 for high IL-6 expression, and <175 for low and ≥175 for high RBP4 expression, respectively.

Gene expression data were obtained from the UCSC Xena platform in the form of RSEM expected counts and subsequently normalized using the DESeq2 package. The initial RNA-seq transcriptomic data were aligned with the STAR aligner and quantified via RSEM. To ensure reliability of the clinicopathological correlations, only patients with colorectal adenocarcinoma and complete clinicopathological information (age, gender, pT, pN, pM, and TNM stage) were included in the analysis, while cases with missing or undefined clinical data were excluded. The final study cohort comprised 350 CRC cases from The Cancer Genome Atlas (TCGA). Expression analyses of *IL-*6 and *RBP4* were conducted based on RNA-seq data sourced from the UCSC Xena Browser (http://xena.ucsc.edu; accessed on 7 July 2025). The DESeq2 median-of-ratios normalization method was employed to correct for differences in sequencing depth and RNA composition among samples. For *IL-6*, a cut-off value of 6.728 was determined, classifying samples into low (<6.728) and high (≥6.728) expression groups. For *RBP4*, a cut-off value of 7.213 was determined, classifying samples into low (<7.213) and high (≥7.213) expression groups.

To identify genes with positive correlation to *IL-6* and *RBP4*, data from the TCGA cohort were analyzed using the cBioPortal platform (https://www.cbioportal.org; accessed on 7 July 2025), extracting the top 50 co-expressed genes for each target. Pathway enrichment analyses were performed with the Reactome Pathway Database (https://reactome.org; accessed on 7 July 2025) and the KEGG Pathway Database (https://www.genome.jp/kegg/pathway.html; accessed on 9 July 2025) to explore molecular mechanisms potentially involved in CRC pathogenesis.

Protein–protein interaction (PPI) networks were constructed for the top IL-6- and RBP4-associated genes using the STRING database (https://string-db.org; accessed on 14 July 2025), applying a medium confidence score threshold (0.700). Network visualizations were generated in Cytoscape software (version 3.10.3, Cytoscape Consortium, San Diego, CA, USA). Functional annotation of co-expressed genes, including classification into Gene Ontology (GO) categories—biological processes (BP), molecular functions (MF), and cellular components (CC)—was conducted using the Database for Annotation, Visualization and Integrated Discovery (DAVID; https://david.ncifcrf.gov; accessed on 9 July 2025).

### 2.4. Statistical Analysis

All statistical analyses and visualizations were performed using GraphPad Prism (version 8.0; GraphPad Software, San Diego, CA, USA) and RStudio (version 1.3.1093). The Shapiro–Wilk test was applied to assess the normality of continuous variables. Based on the distribution of the data, appropriate parametric or non-parametric statistical tests were selected. Comparisons of IL-6 and RBP4 expression levels between tumor tissues and adjacent non-tumorous tissues were conducted using the Mann–Whitney U test. The relationships between categorized IL-6 and RBP4 expression and clinicopathological parameters were analyzed using the chi-square test or Fisher’s exact test, depending on the expected cell counts. Overall survival (OS) was estimated by Kaplan–Meier survival analysis, and differences between survival curves were evaluated with the log-rank test. All *p*-values were two-tailed, and a significance level of *p* < 0.05 was considered statistically significant.

## 3. Results

### 3.1. Immunoexpression of IL-6 and RBP4 in Tumor and Normal Adjacent Tissue and Its Clinicopathological Associations in Our Cohort

**Figure 1:**
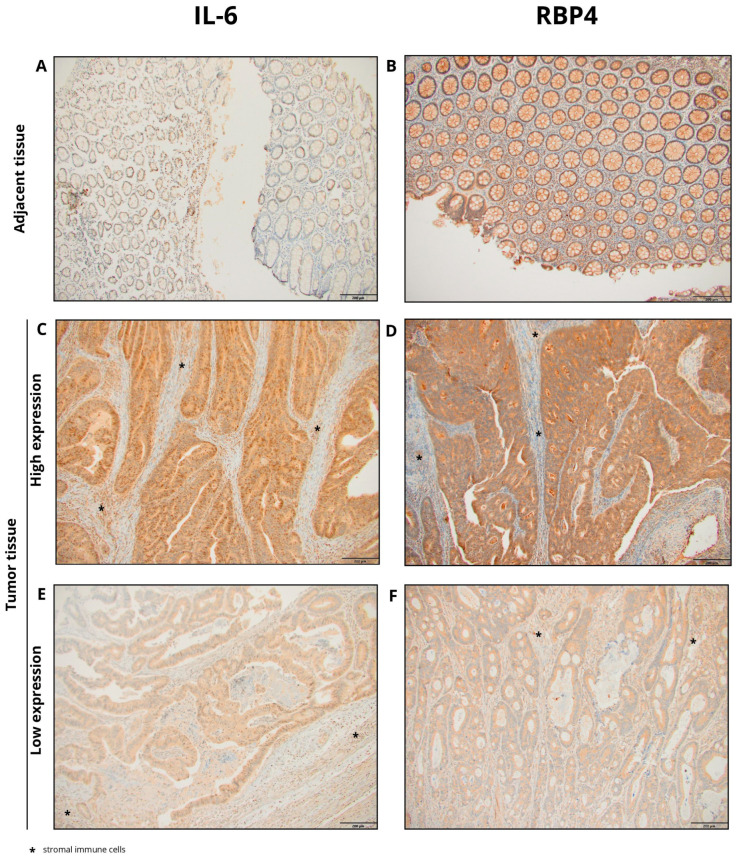
Representative images of immunohistochemical staining for IL-6 and RBP4 in colorectal cancer (CRC) and adjacent normal tissues. (**A**) IL-6 staining in adjacent tissue; (**B**) RBP4 staining in adjacent tissue; (**C**) high IL-6 expression in CRC tissue; (**D**) high RBP4 expression in CRC tissue; (**E**) low IL-6 expression in CRC tissue; (**F**) low RBP4 expression in CRC tissue (primary magnification ×20), * stromal immune cells.

**Figure 2:**
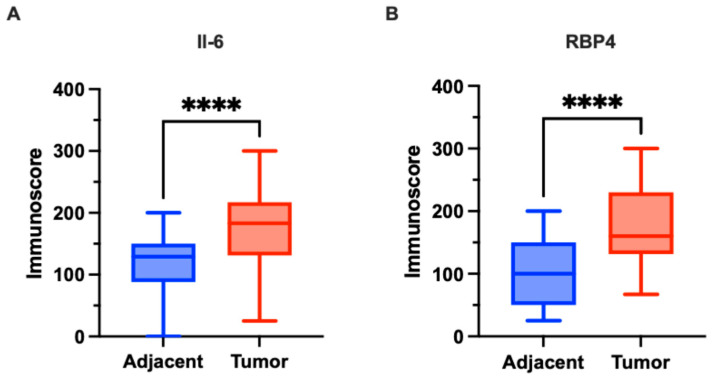
Expression of IL-6 and RBP4 in colorectal cancer (CRC) tissues (*n* = 118) and adjacent normal tissues (*n* = 42) in our cohort. (**A**) IL-6 expression was significantly higher in CRC tissues compared to adjacent normal tissues (*p* < 0.0001). (**B**) RBP4 expression was significantly higher in CRC tissues compared to adjacent normal tissues (*p* < 0.0001). Data are presented as box-and-whisker plots indicating median, interquartile range, and minimum to maximum values. The asterisks (****) indicates *p-*value < 0.0001.

Cytoplasmic expression of IL-6 was observed in CRC tissues. The staining pattern was characterized by diffuse, non-uniform cytoplasmic labeling of tumor cells, with areas of more intense staining, particularly at the invasive front. Additionally, IL-6 expression was noted in stromal immune cells, including macrophages, within the tumor microenvironment. In adjacent non-cancerous tissues, cytoplasmic IL-6 expression was also detected but at markedly lower levels. Representative images illustrating IL-6 immunohistochemical staining in tumor and adjacent tissues are presented in Figure 1. Quantitative analysis revealed significantly higher IL-6 expression in CRC tissues compared to adjacent tissues (median: 183.0 vs. 129.0; *p* < 0.0001; Figure 2A).

Cytoplasmic expression of RBP4 was demonstrated in CRC tissues. The cytoplasmic staining pattern was characterized by ubiquitous, diffuse labeling of tumor cells, although with variable intensity across the tumor tissue. Additionally, RBP4 expression was observed in macrophages present within the tumor microenvironment. In the control group, consisting of adjacent non-cancerous tissues, cytoplasmic RBP4 expression was also detected. Representative images illustrating RBP4 immunohistochemical staining in tumor and adjacent tissues are presented in Figure 1. RBP4 expression was significantly elevated in colorectal cancer (CRC) tissues compared to adjacent non-tumorous tissues (*p* < 0.0001; Figure 2B).

The association between IL-6 protein expression and clinicopathological features was assessed in 118 colorectal cancer (CRC) patients (Table 1). High IL-6 expression was observed in 27 cases (22.9%), while low expression was present in 91 cases (77.1%). No significant associations were identified between IL-6 expression and gender, tumor grade, primary tumor (pT) status, lymph node involvement (pN), distant metastasis (pM), TNM stage, vascular invasion (VI), perineural invasion (PNI), resection margin status, or primary tumor location (all *p* > 0.05). However, significantly higher IL-6 expression was observed in patients aged over 65 years (*p* = 0.0254), in those with BMI ≥ 30 kg/m^2^ (*p* < 0.0001), and in patients with elevated waist circumference (≥88 cm for females, ≥102 cm for males; *p* = 0.0127) (Table 1).

Applying the defined cut-off value, high RBP4 expression was identified in 50 cases (42.37%), while low expression was observed in 68 cases (57.63%). High RBP4 expression was significantly associated with advanced pT status (*p* = 0.0398), positive lymph node involvement (pN1–2; *p* = 0.0044), and advanced TNM stage (III–IV; *p* = 0.0119). Furthermore, patients with obesity (BMI ≥ 30 kg/m^2^) demonstrated significantly higher RBP4 expression compared to non-obese patients (*p* = 0.0264). Patients with elevated waist circumference (≥88 cm in females, ≥102 cm in males) showed a higher frequency of RBP4 overexpression (26 cases, 52%) compared to those with lower waist circumference (16 cases, 33%) (*p* = 0.0396). No significant associations were found between RBP4 expression and age, gender, tumor grade, distant metastasis (pM), vascular or perineural invasion, resection margin status, or primary tumor location (Table 1).

### 3.2. Association with the Clinical Outcome in Our Cohort

**Figure 3:**
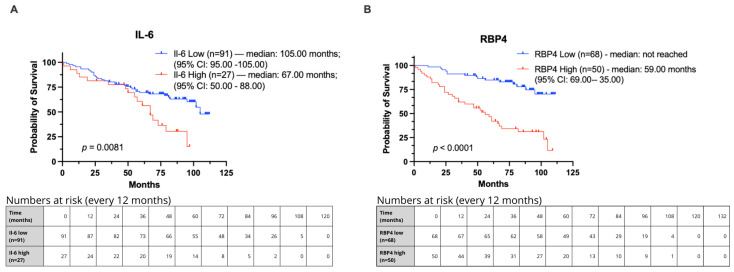
Kaplan–Meier curves showing overall survival in CRC patients from our cohort stratified by IL-6 and RBP4 expression levels. Groups were defined using optimal cut-off points determined by Evaluate Cutpoints (R version 4.4.2). (**A**) Survival comparison for IL-6. (**B**) Survival comparison for RBP4.

In Kaplan–Meier survival analysis of our cohort, IL-6 high expression was significantly associated with shortened median overall survival (OS) of 67 months compared with 105 months in the IL-6 low-expression group (*p* = 0.0081; HR = 2.136, 95% CI: 1.061–4.302; Figure 3A). Similarly, RBP4 high expression was significantly associated with reduced median OS of 59 months, whereas the median OS in the RBP4 low-expression group was not reached (*p* < 0.0001; HR = 4.360, 95% CI: 2.417–7.868; Figure 3B).

### 3.3. Immunoexpression of IL-6 and RBP4 in Tumor and Normal Adjacent Tissue and Its Clinicopathological Associations and Association with the Clinical Outcome of TCGA Cohort

**Figure 4:**
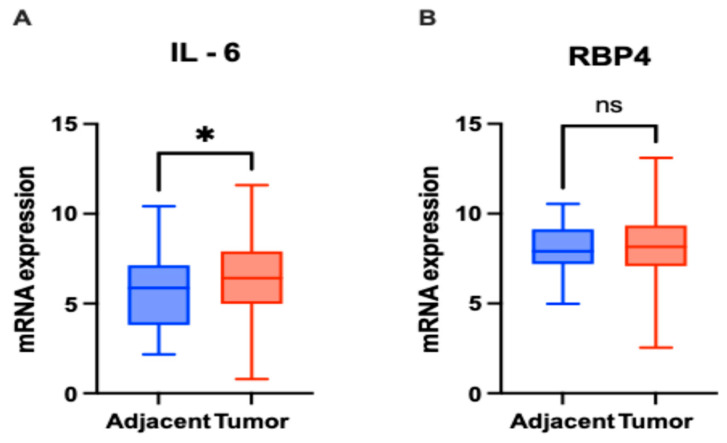
mRNA expression levels of *IL-6* and *RBP4* in colorectal cancer (CRC) tissues (*n* = 363) and adjacent normal tissues (*n* = 51) based on RNA-Seq data. (**A**) *IL-6* expression was significantly higher in CRC tissues compared to adjacent normal tissues (*p* = 0.0243). (**B**) *RBP4* expression showed no significant difference between CRC and adjacent normal tissues (*p* = 0.6338) (ns—not significant). Data are presented as box-and-whisker plots indicating median, interquartile range, and minimum to maximum values. The asterisks (*) indicates *p-*value < 0.01.

In the TCGA cohort, *IL-6* mRNA expression was significantly higher in CRC tissues compared to adjacent normal tissues (*p* = 0.0243; Figure 4A). In contrast, *RBP4* mRNA expression did not show a significant difference between tumor and adjacent tissues (*p* = 0.6338; Figure 4B).

We evaluated whether *IL-6* and *RBP4* mRNA expression levels were associated with clinicopathological parameters of CRC patients (Table 2). Based on the defined cut-offs, 191 (54.57%) patients showed low *IL-6* expression, while 159 (45.43%) demonstrated high *IL-6* expression. Regarding *RBP4* expression, 95 (27.14%) cases were classified as low, and 255 (72.86%) as high. In terms of age, 81 patients aged ≤65 years showed high *IL-6* expression, compared to 78 (43.09%) patients aged >65 years. For *RBP4*, high expression was observed in 121 and 134 patients aged ≤65 and >65 years, respectively. No statistically significant associations were observed for either *IL-6* or *RBP4* expression with age. When examining gender, high *IL-6* expression was observed in 79 males and 80 females. A significant association was found between *RBP4* expression and gender (*p* = 0.0359), with high expression more prevalent among females. Regarding tumor invasion (pT status), high *IL-6* expression was detected in 29 T1–T2, 113 T3, and 17 (36.17%) T4 patients. No significant associations were found for either *IL-*6 or *RBP4* expression with pT status. For nodal status (pN), high *IL-6* expression occurred in 94 N0 and 65 N1–N2 cases. In contrast, *RBP4* expression was significantly associated with pN status (*p* = 0.0018). As for distant metastasis (pM status), high *IL-6* expression was found in 110 M0 and 27 M1 cases. Notably, *RBP4* expression was significantly associated with pM status (*p* = 0.0086), with a lower frequency of high *RBP4* expression in M1 patients. Cases with undefined pM status (MX, *n* = 55) were excluded from the statistical analysis. Finally, when evaluating TNM stage, high *IL-6* expression was observed in 25 stage I, 63 stage II, 48 stage III, and 23 stage IV patients. *RBP4* expression showed a significant association with TNM stage (*p* = 0.0017), with the highest proportion of high expression seen in early-stage patients.

**Figure 5:**
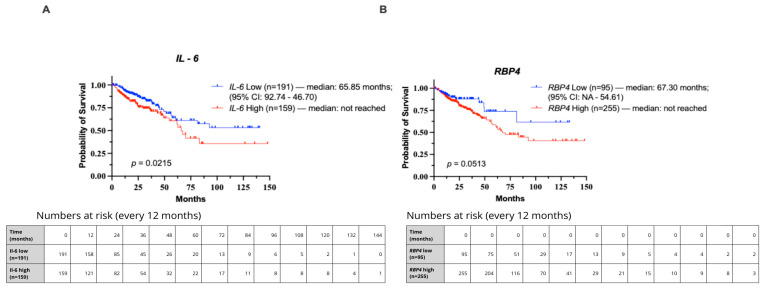
Kaplan–Meier curves showing overall survival in CRC patients from the TCGA cohort stratified by IL-6 and RBP4 expression levels. Groups were defined using optimal cut-off points determined by Evaluate Cutpoints (R version 4.4.2). (**A**) Survival comparison for IL-6. (**B**) Survival comparison for RBP4.

In Kaplan–Meier survival analysis of the TCGA cohort, patients were stratified into low- and high-expression groups for *IL-6* (cut-off = 6.728) and *RBP4* (cut-off = 7.213) using Evaluate Cutpoints software (R version 4.4.2). High *IL-6* expression was significantly associated with poorer overall survival (median overall survival (OS) of 65.85 months compared with not reached) (*p* = 0.0215; HR = 1.654, 95% CI: 1.067–2.563; Figure 5A). For RBP4, high expression showed a trend toward reduced survival, though it did not reach statistical significance (median overall survival (OS) of 67.30 months compared with not reached) (*p* = 0.0513; HR = 1.757, 95% CI: 1.076–2.868; Figure 5B).

### 3.4. Correlation Between IL-6 and RBP4 Expression in Our Cohort and TCGA

Spearman correlation analysis was performed to evaluate the relationship between IL-6 and RBP4 expression. In the TCGA cohort (*n* = 363), a weak but statistically significant positive correlation was observed (r = 0.145, *p* = 0.0056). In the IHC cohort (*n* = 118), no significant correlation between IL-6 and RBP4 protein expression was found (r = 0.043, *p* = 0.646). When the analysis was restricted to patients with abdominal obesity (waist circumference ≥88 cm for females/≥102 cm for males), a stronger positive correlation was identified (r = 0.310, *p* = 0.034).

### 3.5. Univariate and Multivariate Cox Regression Analyses of Prognostic Factors in CRC

As presented in Table 3, statistically significant hazard ratios (HRs) were observed in the univariable analysis for IL-6 (HR 1.94, 95% CI 1.04–3.61, *p* = 0.038), RBP4 (HR 4.58, 95% CI 2.45–8.59, *p* < 0.0001), age (HR 1.05, 95% CI 1.02–1.08, *p* = 0.004), gender (HR 2.58, 95% CI 1.31–5.07, *p* = 0.006), TNM stage (HR 3.45, 95% CI 1.94–6.13, *p* < 0.0001), and lymph node status (HR 3.30, 95% CI 1.85–5.86, *p* < 0.0001). Obesity was not significantly associated with overall survival (HR 1.13, 95% CI 0.61–2.09, *p* = 0.696). In the subsequent multivariable Cox regression model, RBP4 (HR 3.40, 95% CI 1.78–6.51, *p* < 0.0001), TNM stage (HR 3.03, 95% CI 1.66–5.51, *p* < 0.0001), and age (HR 1.03, 95% CI 1.00–1.07, *p* = 0.048) remained independent prognostic factors for overall survival. IL-6 and gender did not retain statistical significance in the multivariable model (*p* = 0.096 and *p* = 0.067, respectively).

In the TCGA cohort, univariable analysis identified IL-6 (HR 0.90, 95% CI 0.82–1.00, *p* = 0.044), age (HR 1.03, 95% CI 1.01–1.05, *p* = 0.002), TNM stage (HR 2.52, 95% CI 1.58–4.02, *p* < 0.0001), and pN status (HR 2.34, 95% CI 1.48–3.70, *p* < 0.0001) as significant predictors of overall survival. RBP4 did not show statistical significance (*p* = 0.773). In the multivariable TCGA analysis, age (HR 1.04, 95% CI 1.02–1.06, *p* < 0.0001) and TNM stage (HR 3.12, 95% CI 1.93–5.03, *p* < 0.0001) remained independent prognostic factors, while IL-6, RBP4, gender, and pN status lost statistical significance (Table 4).

### 3.6. In Silico Investigation of Functional Pathways Connected to RBP4 and IL-6-Related Genes of the TCGA Cohort

Spearman’s rank correlation analysis of CRC data obtained from cBioPortal (https://www.cbioportal.org, accessed on 7 July 2025) identified genes whose expression levels were positively or negatively associated with IL-6 and *RBP4*. Positive correlations indicate genes with expression patterns that increase alongside *IL-6* or *RBP4*, while negative correlations reflect genes whose expression decreases as *IL-6* or *RBP4* expression rises. Among the top 50 genes positively correlated with *IL-6* (Table 5), *IL11* (ρ = 0.765, *p* = 5.83 × 10^−102^) and *SOCS3* (ρ = 0.753, *p* = 9.05 × 10^−97^) showed the strongest associations. For negatively correlated genes (Table 6), HNF1B (ρ = –0.356, *p* = 4.10 × 10^−17^) and *IL17RE* (ρ = –0.356, *p* = 4.39 × 10^−17^) demonstrated the highest inverse relationships. Similarly, for *RBP4, COLEC11* (ρ = 0.369, *p* = 2.19 × 10^−18^) and CDIPT (ρ = 0.368, *p* = 2.95 × 10^−18^) were the most strongly positively correlated genes (Table 7), while *HSPA4L* and *PPAT* again emerged as the strongest negative correlations (ρ =−0.398, *p* = 2.71 × 10^−21^ and ρ = –0.365, *p* = 6.35 × 10^−18^, respectively; Table 8).

**Figure 6:**
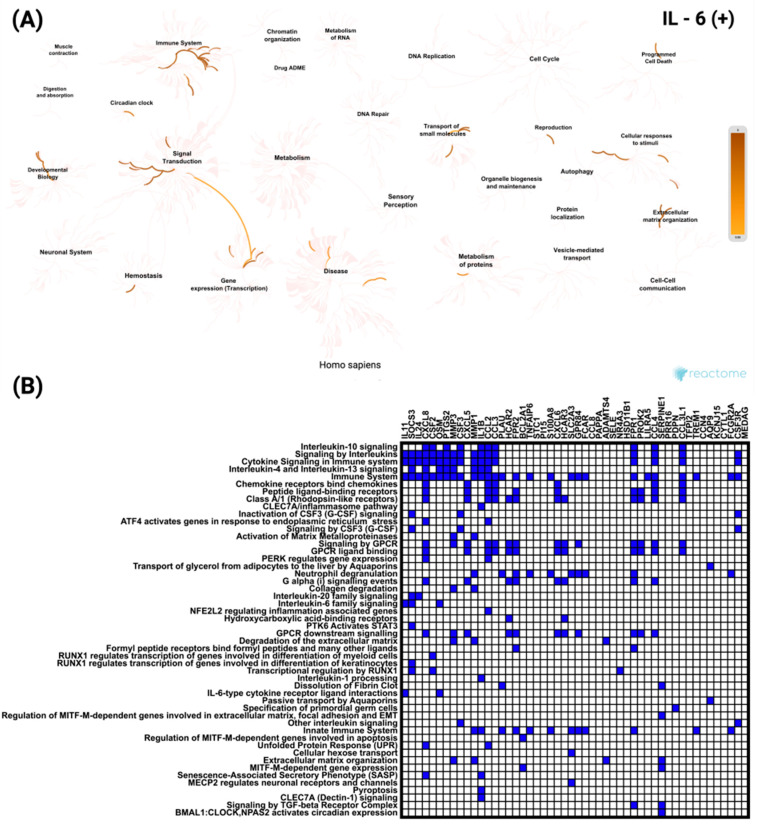
Functional enrichment analysis of the top 50 genes most significantly positively correlated with *IL-6* in CRC based on Reactome pathways. (**A**) Network visualization showing the most significantly enriched biological processes and pathways associated with *IL-6* correlated genes. Color intensity indicates the degree of enrichment. (**B**) Heatmap illustrates the involvement of these genes in the most significant Reactome pathways, highlighting their distribution across diverse cellular and molecular processes.

Functional enrichment analysis of the top 50 genes most significantly positively correlated with *IL-6* in CRC was performed using Reactome (accessed on 7 July 2025). In Figure 6A, the network visualization highlights strong enrichment for multiple immune-related pathways. All these pathways showed the same level of statistical significance (*p* < 1.1 × 10^−16^). In Figure 6B, the heatmap illustrates the distribution of individual genes across these pathways, with *CXCL8* (*Interleukin-8*) being the most frequently represented, emphasizing its central role in immune system regulation.

**Figure 7:**
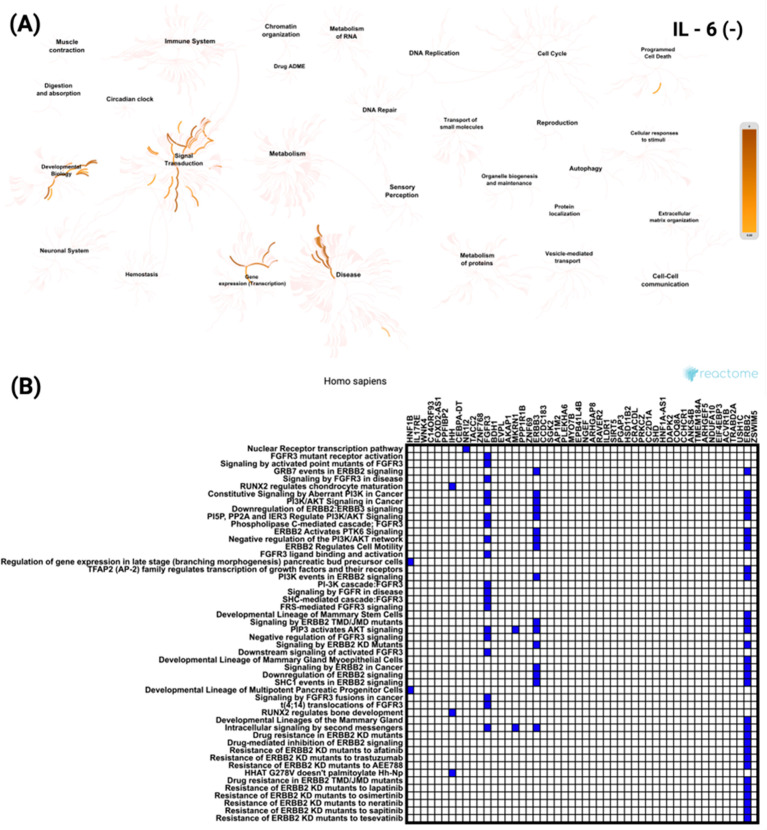
Functional enrichment analysis of the top 50 most significantly negatively correlated genes with *IL-6* in CRC based on Reactome pathways. (**A**) Network visualization presenting the most significantly enriched biological processes and pathways linked to *IL-6* negatively correlated genes. The color intensity reflects the strength of enrichment. (**B**) Heatmap showing the distribution of these genes across the most significant Reactome pathways, emphasizing their roles in various cellular and molecular functions.

In contrast, analysis of the top 50 genes negatively correlated with *IL-6* revealed enrichment for signaling and transcriptional pathways unrelated to immune processes (Figure 7A). These included the Nuclear Receptor transcription pathway and *FGFR3* mutant receptor activation. As shown in Figure 7B, *ERBB2* was the most frequently involved gene among the negatively correlated set, appearing across multiple enriched pathways. All these pathways reached the same level of statistical significance (*p* < 5.37 × 10^−8^).

**Figure 8:**
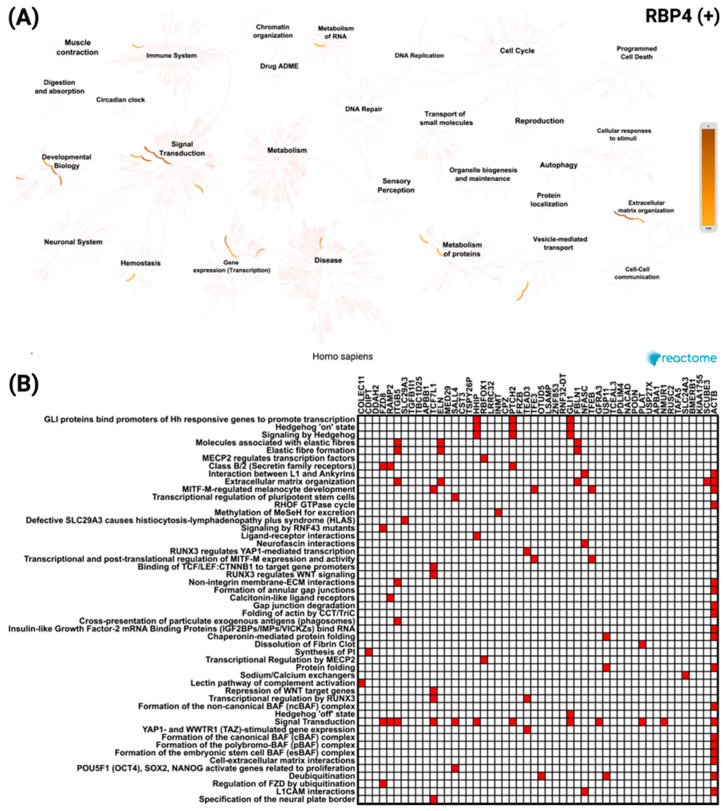
Reactome pathway enrichment analysis of the top 50 genes most significantly positively correlated with *RBP4* in CRC. (**A**) Network diagram showing the key biological processes and pathways enriched among *RBP4* positively correlated genes, with color intensity indicating enrichment strength. (**B**) Heatmap displaying the distribution of these genes across the most significantly enriched Reactome pathways, reflecting their association with diverse cellular and molecular functions.

To further explore these associations, a similar analysis was performed for *RBP4*-correlated genes. For the top 50 positively correlated genes, the network visualization (Figure 8A) demonstrated enrichment predominantly in signal transduction and developmental biology pathways. The most significant pathway was *GLI* proteins binding to promoters of *Hh*-responsive genes to promote transcription, with a *p* value of 2.38 × 10^−8^. The heatmap (Figure 8B) revealed *ACTB* as the most frequently represented gene across these enriched categories.

**Figure 9:**
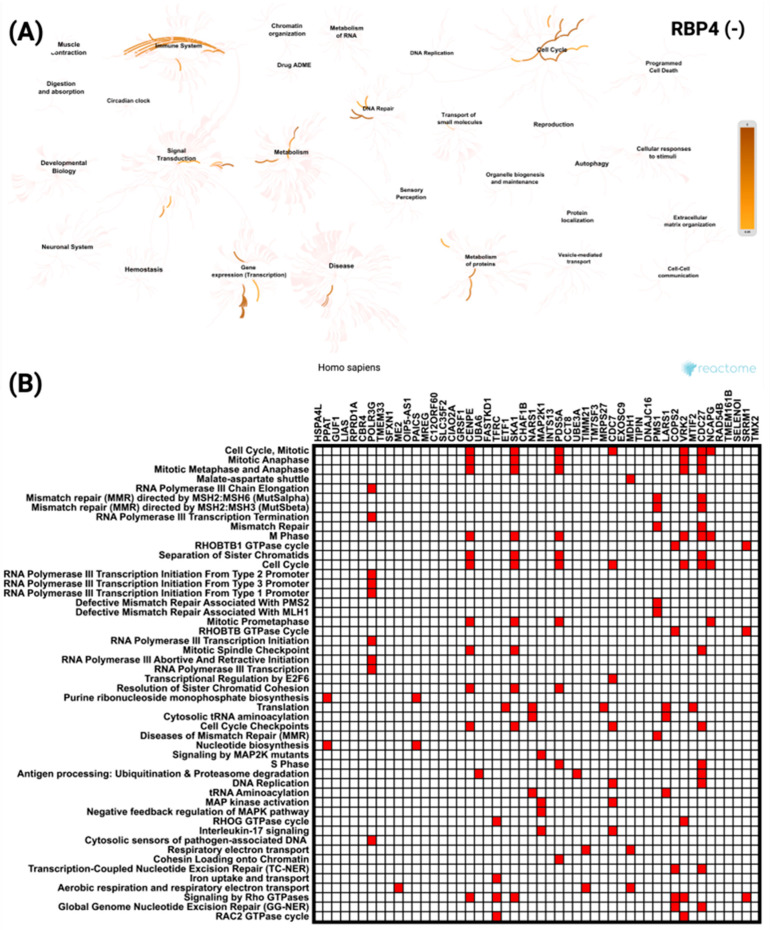
Reactome pathway analysis of the top 50 genes most significantly negatively correlated with *RBP4* in CRC. (**A**) Network visualization highlighting the key biological processes and pathways enriched among RBP4 negatively correlated genes, with color intensity representing the level of enrichment. (**B**) Heatmap showing the distribution of these genes across the most significantly enriched Reactome pathways, indicating their involvement in a range of cellular and molecular processes.

Meanwhile, the top 50 negatively correlated genes showed enrichment mainly in pathways related to cell cycle and immune system processes (Figure 9A). The most significant pathways included Cell Cycle Mitotic (*p* = 0.0012) and Mitotic Anaphase (*p* = 0.0016). The heatmap (Figure 9B) identified *CDC27* as the most frequently represented gene within these pathways.

**Figure 10:**
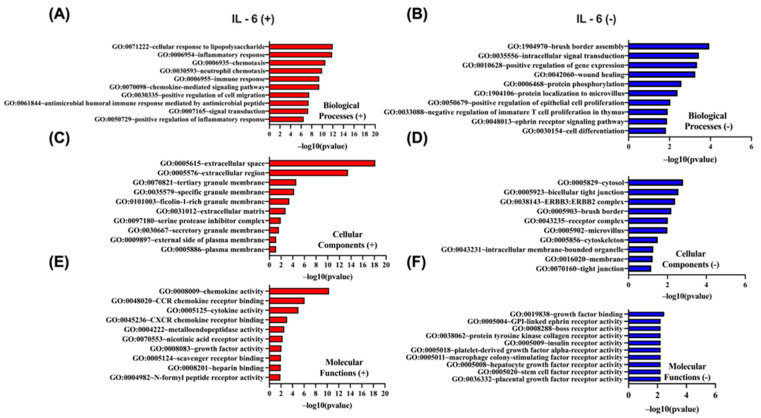
GO enrichment analysis of the top 50 genes most significantly positively and negatively correlated with *IL-6* in CRC using DAVID. GO terms were classified into biological processes (BP), cellular components (CC), and molecular functions (MF). The top 10 enriched GO terms for each category are presented, ranked by −log10(*p*-value). (**A**,**C**,**E**) display BP, CC, and MF for positively correlated genes, respectively, while (**B**,**D**,**F**) show BP, CC, and MF for negatively correlated genes.

A parallel Gene Ontology enrichment analysis was carried out for genes whose expression correlates with *IL-6* using the DAVID tool (https://davidbioinformatics.nih.gov/home.jsp, accessed on 9 July 2025). Genes positively correlated with *IL-6* (Figure 10A,C,E) are most significantly enriched for the cellular response to lipopolysaccharide (GO:0071222), localize predominantly to the extracellular space (GO:0005615), and exhibit chemokine activity (GO:0008009). In contrast, genes negatively correlated with *IL-6* (Figure 10B,D,F) are dominated by brush border assembly (GO:1904970) among biological processes, cytosolic localization (GO:0005829) as their principal cellular component, and growth factor binding (GO:0019838) as their top molecular function.

**Figure 11:**
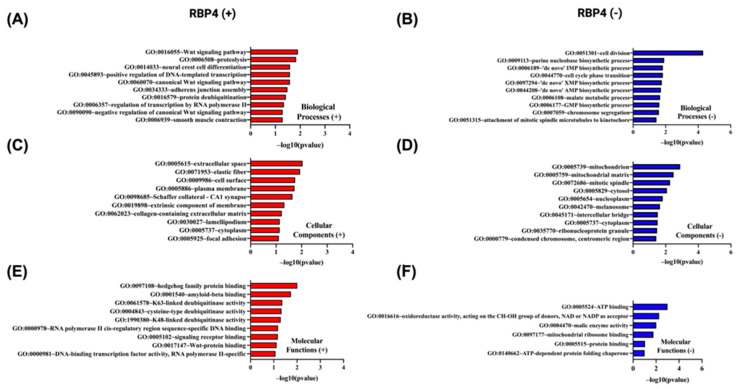
Gene Ontology (GO) enrichment analysis of the top 50 genes most significantly positively and negatively correlated with *RBP4* in CRC using DAVID. GO terms were categorized into biological processes (BP), cellular components (CC), and molecular functions (MF). The top 10 enriched GO terms for each category are shown, ranked by −log10(*p*-value). (**A**,**C**,**E**) represent BP, CC, and MF for positively correlated genes, respectively. (**B**,**D**,**F**) represent BP, CC, and MF for negatively correlated genes, respectively.

In Figure 11, Gene Ontology enrichment analysis of proteins whose abundance correlates positively (Figure 11A,C,E) or negatively (Figure 11B,D,F) with *RBP4* gene expression was performed using the DAVID tool (https://davidbioinformatics.nih.gov/home.jsp, accessed on 9 July 2025). Among the proteins up-regulated in parallel with *RBP4*, the Wnt signaling pathway (GO:0016055) is the most significant biological process (Figure 11A), the extracellular space (GO:0005615) is the predominant cellular component (Figure 11C), and hedgehog family protein binding (GO:0097108) is the top molecular function (11E). By contrast, proteins whose levels decrease as RBP4 expression rises are most strongly enriched for cell division (GO:0051301) as a biological process (Figure 11B), localization to the mitochondrion (GO:0005739) as a cellular component (Figure 11D), and ATP binding (GO:0005524) as a molecular function (Figure 11F). Together, these data indicate that proteins co-regulated with *RBP4* partition into an extracellular signaling/developmental module versus a proliferation/mitochondrial energetics module.

**Figure 12:**
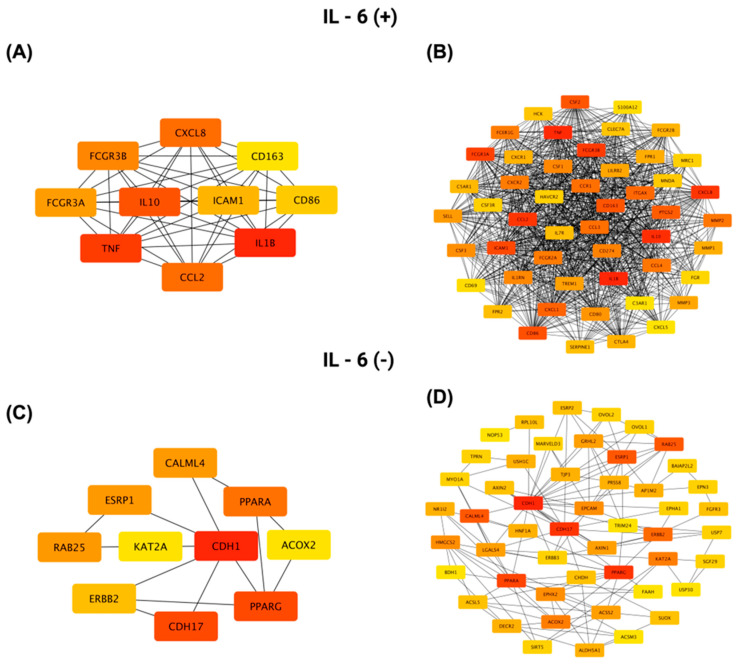
Protein–protein interaction (PPI) network analysis of the top 50 genes most significantly positively and negatively correlated with *IL-6* in CRC, generated using Cytoscape. (**A**,**C**) Networks highlighting the 10 genes with the highest connectivity (determined by degree centrality) within the positively and negatively correlated gene sets, respectively. (**B**,**D**) Comprehensive PPI networks showing all 50 positively (**B**) and negatively (**D**) correlated genes and their interaction patterns. Node color indicates connectivity level, with red representing a higher degree and yellow a lower degree.

A protein-protein interaction (PPI) network was constructed for genes positively correlated with *IL-6* using STRING (https://string-db.org; accessed on 14 July 2025) and visualized in Cytoscape v3.10.3. Figure 12A shows the top ten hub genes ranked by connectivity in the *IL-6* positive network, with *IL1B*, *TNF,* and *IL10* as the three most connected nodes. Figure 12B presents the complete interactome of 50 nodes and 859 edges. This network has an average of 34.36 neighbors per node, a clustering coefficient of 0.819, a network density of 0.701, a diameter of 3, a radius of 2, and a characteristic path length of 1.300. It exhibits highly significant protein interaction enrichment (*p* < 1 × 10^−16^).

For genes negatively correlated with *IL-6*, Figure 12C highlights the top ten hub genes, led by *CDH1*, *PPARG,* and *CDH17*, while Figure 12D shows the full network of 50 nodes connected by 144 edges. This network has an average of 5.76 neighbors per node, a clustering coefficient of 0.517, a network density of 0.118, a diameter of 5, a radius of 3, and a characteristic path length of 2.663, reflecting a more modular architecture centered on epithelial adhesion and differentiation pathways.

**Figure 13:**
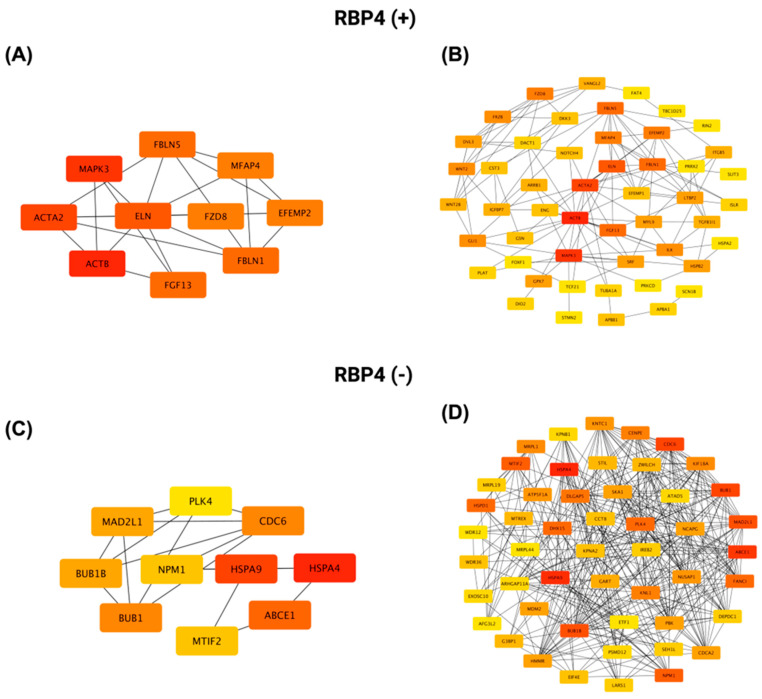
PPI network analysis of the top 50 genes most significantly positively and negatively correlated with *RBP4* in CRC using Cytoscape. (**A**,**C**) Networks showing the top 10 most highly connected genes (based on degree centrality) among the positively and negatively correlated gene sets, respectively. (**B**,**D**) Full PPI networks of all 50 positively (**B**) and negatively (**D**) correlated genes, illustrating the overall interaction landscape. Node color reflects connectivity, with red indicating a higher degree and yellow a lower degree.

Similarly, a PPI network was constructed for proteins positively correlated with *RBP4* using STRING (https://string-db.org; accessed 14 July 2025) and visualized in Cytoscape v3.10.3. Figure 13A shows the top ten hub proteins ranked by connectivity in the *RBP4* positive network, with *ACTB*, *MAPK3,* and *ACTA2* as the three most connected nodes. Figure 13B presents the complete interactome of 50 nodes and 130 edges. This network has an average of 5.31 neighbors per node, a clustering coefficient of 0.410, a network density of 0.111, a diameter of 7, a radius of 4, and a characteristic path length of 2.771.

For proteins negatively correlated with *RBP4*, Figure 13C highlights the top ten hub proteins, led by *HSPA4*, *HSPA9,* and *ABCE1*, while Figure 13D shows the full network of 50 nodes connected by 353 edges. This network has an average of 14.12 neighbors per node, a clustering coefficient of 0.653, a network density of 0.288, a diameter of 4, a radius of 2, and a characteristic path length of 2.109.

## 4. Discussion

Colorectal cancer is a major public health concern due to its high rate of occurrence and the fact that its symptoms are often non-specific, making diagnosis challenging [2,3,4]. For this reason, in recent years, numerous studies have been conducted to identify new biomarkers involved in the pathogenesis and progression of colorectal cancer. Recent discoveries in this area have drawn researchers’ attention to the link between tumorigenesis, obesity, and inflammation as important drivers of colorectal cancer [10,11,12,18,19,21,24,28,29,30,31]. We believed that this line of research was necessary and should continue. Thus, we selected IL-6 as the factor connecting inflammation to the growth of colorectal cancer and RBP4 as the link between obesity and tumorigenesis.

IL-6 is a pro-inflammatory cytokine that has been shown to induce acute phase responses, act as a maturation factor for B lymphocytes, participate in T lymphocyte differentiation, and activate natural killer (NK) cells [6,7]. Elevated levels of IL-6 are indicative of inflammation, a common occurrence in cancerous diseases. The initiation of the JAK/STAT3, Ras/MAPK, and PI3K/Akt pathways results in the regulation of gene products that stimulate proliferation, angiogenesis, or metastasis. Its role has been described in a variety of neoplasms, including multiple myeloma, renal cell carcinoma, cervical cancer, and prostate cancer, among others [7,11]. The presence of CEA in colorectal cancer has been demonstrated to stimulate the secretion of IL-6 by Kupffer cells. This, in turn, promotes colorectal cancer metastasis to the liver [8,9]. IL-6 stimulates the secretion of HGF (hepatocyte growth factor), which increases cell motogenic activity and also promotes metastasis formation [9,10]. Furthermore, a correlation with a worse prognosis has been observed, which may be attributable to the inhibition of the anti-tumor response by IL-6 [9,12].

The present study demonstrated that the expression of interleukin-6 (IL-6) in the tumor tissue was significantly higher than in the adjacent tissue. In both tumor and adjacent tissue, the sites of highest IL-6 expression were cancer cells and inflammatory cells infiltrating the tumor, but to a lesser extent, expression also appeared in fibroblasts in the lining. This finding aligns with most reports on IL-6 expression [8,9,10,11,14,30,31,32,33,34]. Notably, the results obtained from immunohistochemical evaluation were further corroborated by in silico analyses of transcriptomic data derived from The Cancer Genome Atlas (TCGA), which confirmed increased IL-6 mRNA expression in colorectal cancer tissue compared to adjacent normal mucosa. These concordant findings strengthen the evidence for IL-6 upregulation in CRC and underscore its potential involvement in tumor-related inflammation.

In our study, a statistically significant association was observed between IL-6 expression and patient age, with higher expression levels noted in individuals over 65 years. This finding aligns with the concept of “inflammaging”, which refers to age-related chronic low-grade inflammation and has been widely associated with increased circulating IL-6 levels. Although previous reports have demonstrated a general trend of elevated IL-6 with advancing age, the relationship is not universally consistent and may be influenced by factors such as comorbidities, gender, or immune status. Our findings support the hypothesis that aging contributes to a pro-inflammatory tumor microenvironment, which may in turn influence cancer progression [35]. A comparable absence of significant associations between IL-6 expression and clinicopathological parameters—including gender, tumor location, histological subtype, and tumor stage—was also demonstrated by Lu et al., further supporting the heterogeneity of IL-6 involvement in colorectal cancer pathophysiology [31]. In turn, results from other authors have shown that higher IL-6 expression correlates with metastasis [10,36], angiogenesis [32], greater tumor cell proliferation [32], and larger tumor size [9,33]. This phenomenon can be attributed, at least in part, to the role of inflammation in stimulating angiogenesis by inducing the expression of angiogenesis-related proteins such as VEGFA [32]. In addition, IL-6 autocrine increases the motogenic activity of tumor cells by binding to the receptor for IL-6 on the surface of tumor cells and increases the secretion of HGF, which promotes metastasis [10,11]. In the in silico analysis assessing the relationship between IL-6 mRNA expression and clinicopathological variables such as age, sex, pT, pN, pM status, and TNM stage, no statistically significant associations were identified. Discrepancies between the findings of different studies may be attributed to variations in cohort size, characteristics of the studied populations, and the inherent heterogeneity of colorectal cancer. This observation aligns with the findings of Legrand-Poels et al., who reported inconsistent IL-6 expression due to promoter suppression [37]. Moreover, some studies suggest that IL-6 expression may decrease in advanced-stage tumors, potentially reflecting adaptive changes in the tumor microenvironment [30]. In our study, significantly higher IL-6 expression was observed in colorectal cancer (CRC) patients with obesity (BMI ≥ 30 kg/m^2^) and increased waist circumference. This finding is consistent with the report by Lara Kern et al., who described IL-6 as a pleiotropic cytokine playing a central role in the crosstalk between metabolic dysfunction and inflammation. Elevated IL-6 levels in obesity contribute to a chronic low-grade inflammatory state that supports tumor development and progression. IL-6 exerts its effects through the activation of several oncogenic pathways, including JAK/STAT3, PI3K/Akt, and MAPK, which promote tumor cell proliferation, inhibit apoptosis, and enhance metastatic potential. In the context of CRC, our findings suggest that obesity-related upregulation of IL-6 may contribute to the creation of a tumor-promoting microenvironment. These results highlight the importance of IL-6 as a molecular link between obesity and colorectal carcinogenesis, emphasizing its potential value as a prognostic biomarker or therapeutic target [38].

In our Kaplan–Meier survival analysis, a significant association was observed between IL-6 expression and overall survival in patients with colorectal cancer (CRC). Patients with high IL-6 expression exhibited shorter survival times compared to those with low expression, both at the protein and mRNA levels. Similarly, Olsen et al. (2015) reported that elevated IL-6 transcript levels in colon carcinoma tissues were significantly associated with poorer overall survival [38]. This finding is further supported by other studies demonstrating that IL-6 contributes to worse prognosis through activation of STAT3 via the gp130 subunit. STAT3 promotes cell cycle progression through the G1 and G2/M phases, thereby stimulating tumor cell proliferation and prolonging their survival. These mechanisms position IL-6 as a tumor-promoting cytokine. Conversely, the absence of IL-6 receptor expression, which prevents cytokine-receptor binding, has been associated with longer survival and more favorable outcomes. This relationship has been documented not only in CRC but also in other malignancies, including esophageal cancer, multiple myeloma, and Kaposi’s sarcoma [11,14].

RBP4 is a retinol-binding protein secreted by adipocytes that has been shown to transport retinol to extrahepatic tissue [15,16,17]. In addition to adipose tissue and liver, it is also synthesized to a lesser extent by the lungs, brain, and kidneys [16]. RBP4 activates STRA6, leading to activation of Janus kinase and STAT3/STAT5, which results in pro-inflammatory response [17,39] and tumorogenesis [19].This results in cancer progression, migrations, and metastasis [17,18,19]. In addition, elevated RBP4 expression has been mentioned in ovarian cancer [17,40], breast cancer [39,41], and pancreatic cancer [21,23]. RBP4 levels have been shown to increase with increasing body weight [16] and greater distribution of central adipose tissue [25], which may make them a link between obesity and tumorigenesis [16].

The study revealed that RBP4 expression levels were higher in tumor tissues compared to adjacent non-cancerous tissues. A strong cytoplasmic staining pattern was observed in tumor cells, as well as in the epithelial cells of normal colon tissue. Notably, during the evaluation of RBP4 in colorectal cancer patients, positively stained macrophages were also observed within the tumor microenvironment. This observation adds an additional layer to our understanding of RBP4 distribution and aligns with previous reports highlighting the role of RBP4 in various malignancies [15,17,18,19,29,39,42]. Interestingly, Zhang et al. also reported strong RBP4 expression in immune cells, particularly macrophages [18]. Interestingly, our immunohistochemical findings contrast with TCGA transcriptomic data, which did not demonstrate significant differences in RBP4 mRNA levels between colorectal tumors and adjacent tissues. Such discrepancies between mRNA and protein levels have been widely documented and may arise from differences in transcript and protein stability, post-transcriptional regulation, and the influence of the tumor microenvironment on protein accumulation. However, Zhao et al. (2025) analyzed TCGA/GTEx RNA-seq (COAD/READ) and found significantly higher *RBP4* mRNA in colorectal tumors than in normal mucosa, using log2-transformed counts and standard statistical testing [43]. They complemented this with RT-qPCR/IHC validations, confirming increased intratumoral expression. Together, these independent results strengthen the interpretation that elevated, tumor-intrinsic *RBP4* expression contributes to CRC progression [43].

Our study demonstrated that high RBP4 expression in colorectal cancer (CRC) tissues was significantly associated with pT status, lymph node metastasis, and advanced clinical stage according to the TNM classification. Additionally, these findings were corroborated by in silico analysis, which revealed a significant association between RBP4 expression and lymph node involvement, distant metastases, and TNM staging. The association between RBP4 and tumor size suggests that RBP4 may promote tumor growth, as also described in ovarian cancer, where RBP4 enhances migration and proliferation by activating RhoA/Rock1 and ERK pathways and upregulating MMP2 and MMP9 [17]. The link between RBP4 and lymph node metastasis aligns with reports from hepatocellular carcinoma, where elevated RBP4 levels were associated with increased cell proliferation, invasiveness, and worse prognosis and TNM staging [28]. The observed relationship between RBP4 and TNM staging in CRC is consistent with studies indicating that higher RBP4 serum levels are linked to advanced disease and poor survival, also in breast cancer, where RBP4 was shown to correlate with ER- and PR-negative status, which results in a more unfavorable prognosis [41]. The binding of RBP4 to STRA6 activates JAK, which phosphorylates STATs. STATs activate cell cycle regulatory genes in the nucleus. This results in tumor progression and an increased risk of metastasis [15,16,18]. Inflammation and consequent endothelial dysfunction induced by stimulation of the expression of pro-inflammatory molecules, such as TGF-beta, by RBP4 may also be involved in metastasis formation [44]. Reduction in RBP4 levels results in inactivation of N-cadherin, MMP2, -3, -9, and an increase in E-cadherin, which also proves the roles of RBP4 in cancer cell migration [20].

Furthermore, the significant correlation between RBP4 expression and obesity-related measures in our study supports the well-established role of RBP4 as an adipokine reflecting adipose tissue mass and visceral fat distribution [25]. Because RBP4 is secreted by adipocytes, more adipose tissue results in overexpression of RBP4, and this may affect tumor progression [17]. In addition, RBP4 can activate macrophages through the adipose tissue exosome [28], which increases inflammation that enhances tumorigenesis. RBP4 has been implicated in insulin resistance and chronic low-grade inflammation, both of which are recognized contributors to cancer development and progression [16,17,19,23,24]. RBP4 has been demonstrated to decrease phosphoinositide-3-kinase activity in muscle and increase phosphoenolpyruvate carboxykinase activity. It increases the expression of GLUT4, which inhibits glucose uptake by muscle and adipocytes [19]. Moreover, STAT’s target gene is SOCS3, which inhibits insulin receptor signaling, which also results in insulin resistance [16]. However, the role of RBP4 in the development of insulin resistance and tumorigenesis is less significant in patients with lower BMI, which may be attributable to adipose tissue dysfunction in obesity [19,41].

Our Kaplan–Meier survival analysis in the TCGA cohort revealed a trend suggesting an association between RBP4 expression levels and overall survival in patients with colorectal cancer (CRC). In our own patient cohort, individuals with elevated RBP4 expression demonstrated a shorter survival time compared to those with lower expression levels of this protein. It was reflected in the other reports demonstrating that enhanced RBP4 correlates with unfavorable prognosis in gastric carcinoma [20], hepatocellular carcinoma [28], and glioblastoma [45]. Also, breast cancer showed higher RBP4 expression [41]. In ovarian cancer, RBP4 overexpression stimulates the expression of MMP-2 and MMP-9, which increases the migration of tumor cells and affects the unfavorable prognosis [17].

In the subsequent findings of our study, we demonstrated that the genes *IL11* and *SOCS3* have been identified as top positively correlated with *IL-6* in CRC. This correlation is primarily linked to the activation of the *IL-6/STAT3* signaling pathway, which plays a crucial role in tumor progression and prognosis in CRC [46,47]. When *IL-6* increases in CRC, we observe a fall in *HNF1B* and *IL17RE* expression. This is biologically plausible; *HNF1B* is frequently reduced (and sometimes promoter-methylated) in colorectal carcinomas and low levels associate with recurrence and shorter disease-free survival, indicating a tumor-suppressive role [48]. There is little published specifically on *IL17RE* in CRC. In contrast, studies on *IL17* itself report that it can promote colorectal tumorigenesis through several pathways and is associated with metastasis and poor prognosis, although some data also suggest a protective role [49]. Reactome analysis shows that genes positively correlated with *IL-6* cluster in immune and inflammatory pathways, with *CXCL8* most frequently represented, underscoring an *IL-6/IL-8*-driven inflammatory microenvironment in CRC. In contrast, negatively correlated genes are enriched in non-immune signaling and transcriptional pathways, with *ERBB2* appearing most often, suggesting attenuation of epithelial growth–related programs when *IL-6–*mediated inflammation is dominant. Rising *IL-6* aligns with parallel upregulation of immune genes and reciprocal downregulation of alternative signaling axes, highlighting a functional dichotomy in CRC biology [50].

Within the set of genes correlated with *RBP4*, *COLEC11* (*CL-11*) ranked among the top positive hits; given that *CL-11* drives cancer cell proliferation and tumor growth and its elevated expression associates with advanced stage and poorer survival in CRC, this positive correlation in our data points to a shared pro-tumorigenic program [51,52]. For *CDIPT*, there are essentially no published data on colorectal cancer; its positive correlation with *RBP4* in our set, therefore, represents a novel observation that may reflect shared lipid/inositol metabolism but remains to be experimentally validated. Reactome analysis shows that *RBP4*, genes positively correlated with its expression cluster in signal transduction and developmental pathways, are dominated by the GLI/Hedgehog transcriptional module. Genes negatively correlated with *RBP4* fall into cell cycle and immune system pathways. This divergence suggests that *RBP4*-high tumors prioritize developmental signaling programs, while proliferative and immune processes are comparatively reduced. This inverse relationship suggests that reduced RBP4 expression may release constraints on cell cycle progression, potentially facilitating uncontrolled proliferation and driving CRC progression. Such a mechanism highlights the relevance of RBP4 as a possible modulator of cell division dynamics within the tumor context.

## 5. Conclusions

This study provides comprehensive insights into the role of IL-6 and RBP4 in colorectal cancer (CRC). Both proteins were found to be significantly overexpressed in tumor tissues compared to adjacent non-cancerous mucosa. Elevated IL-6 expression was associated with older age and obesity-related parameters, while RBP4 overexpression correlated with tumor size, lymph node metastasis, advanced TNM stage, and markers of obesity, such as increased BMI and waist circumference. Kaplan–Meier survival analysis demonstrated that high expression of IL-6 and RBP4 tended to associate with shorter overall survival, supporting their potential prognostic relevance. In silico analyses confirmed the transcriptional upregulation of IL6 and RBP4 in CRC and revealed distinct molecular pathways—immune/inflammatory signaling for IL-6 and developmental signaling for RBP4—highlighting their possible contributions to tumor biology.

Taken together, our findings suggest that IL-6 and RBP4 may function as biomarkers of poor prognosis and may represent potential therapeutic targets, particularly in CRC cases linked to inflammation and obesity. Further research is warranted to explore their mechanistic roles and validate their clinical utility in larger, independent cohorts.

## Figures and Tables

**Table 1:** Association of IL-6 and RBP4 protein expression with clinicopathological parameters of colorectal cancer patients.

Clinicopathological Parameters	*n* (%) *n* = 118	Il-6 Expression		*p* Value	RBP4 Expression		*p* Value
		Low	High		Low	High	
		*n* = 91	*n* = 27		*n* = 68	*n* = 50	
Age (years)							
≤65	50 (42.37)	44 (88.00)	6 (12.00)	**0.0254**	34 (68.00)	16 (32.00)	0.0607
>65	68 (57.63)	47 (69.12)	21 (30.88)		34 (50.00)	34 (50.00)	
Gender							
Male	71	54	17	0.8248	36	35	0.0864
Female	47	37	10		32	15	
Grading							
G1–G2	113	86	27	0.5876	63	50	0.0717
G3	5	5	0		5	0	
pT status							
T1–T2	61	49	12		40	21	
T3	54	39	15	0.2757	26	28	**0.0398**
T4	3	2	1		1	2	
pN status							
N0	80	59	21	0.8005	53	27	**0.0044**
N1–N2	35	21	6		13	22	
pM status							
M0	115	88	27	0.5599	67	48	0.5733
M1	3	2	1		1	2	
TNM stage							
I	51	40	11	0.9851	34	17	**0.0119**
II	28	18	10		18	10	
III	33	27	6		13	20	
IV	3	2	1		1	2	
VI							
Absent	105	80	25	0.7298	64	41	0.0708
Present	13	11	2		4	9	
PNI							
Absent	111	87	24	0.1955	66	45	0.1323
Present	7	4	3		2	5	
Resection margin							
R0	114	89	25	0.2243	66	48	>0.9999
R1–R2	4	2	2		2	2	
Primary tumor location							
Right colon	40	30	10	0.9018	26	14	0.3779
Left colon	40	33	7		21	19	
Rectum	38	28	10		21	17	
Obesity							
BMI <30 kg/m^2^	83	74	9	**<0.0001**	48	35	**0.0264**
BMI ≥30 kg/m^2^	35	17	18		12	23	
Waist circumference							
<88 cm (female)/<102 cm (male)	48	40	8	**0.0127**	32	16	**0.0396**
≥88 cm (female)/≥102 cm (male)	47	28	19		21	26	

Abbreviations: VI—vascular invasion, PNI—perineural invasion, BMI—Body Mass Index. TNM stage is based on the AJCC 8th edition. Cases with undefined pN status (NX, *n* = 3), TNM stage (TNMX, *n* = 3), or waist circumference (*n* = 23) were excluded from the statistical analyses. Significant values are in bold.

**Table 2:** Association of *IL-6* and *RBP4* mRNA expression with clinicopathological parameters of colorectal cancer patients.

Clinicopathological Parameters	*n* (%) *n =* 350	*Il-6* Expression		*p* Value	*RBP4* Expression		*p Value*
		Low	High		Low	High	
		*n* = 191	*n* = 159		*n* = 95	*n* = 255	
Age (years)							
≤65	169 (48.29)	88 (52.07)	81 (47.93)	0.364	48 (28.4)	121 (71.6)	0.6086
>65	181 (51.71)	103 (56.91)	78 (43.09)		47 (25.97)	134 (74.03)	
Gender							
Male	189 (54)	110 (58.2)	79 (41.8)	0.1395	60 (31.75)	129 (68.25)	**0.0359**
Female	161 (46)	81 (50.31)	80 (49.69)		35 (21.74)	126 (78.26)	
pT status							
T1–T2	64 (18.29)	35 (54.69)	29 (45.31)	0.3761	17 (26.56)	47 (73.44)	0.9912
T3	239 (68.29)	126 (52.72)	113 (47.28)		65 (27.2)	174 (72.8)	
T4	47 (13.43)	30 (63.83)	17 (36.17)		13 (27.66)	34 (72.34)	
pN status							
N0	195 (55.71)	101 (51.79)	94 (48.21)	0.2386	40 (20.51)	155 (79.49)	**0.0018**
N1–N2	155 (44.29)	90 (58.06)	65 (41.94)		55 (35.48)	100 (54.52)	
pM status							
M0	247 (70.57)	137 (55.47)	110 (44.53)	0.1364	62 (25.1)	185 (74.9)	**0.0086**
M1	48 (13.71)	21 (43.75)	27 (56.25)		21 (43.75)	27 (56.25)	
MX	55 (15.71)	33 (60)	22 (40)		12 (21.82)	43 (78.18)	
TNM stage							
I	56 (16)	31 (55.36)	25 (44.64)	0.9259	15 (26.79)	41 (73.21)	**0.0017**
II	132 (37.71)	69 (52.27)	63 (47.73)		22 (16.67)	110 (83.33)	
III	110 (31.43)	62 (56.36)	48 (43.64)		36 (32.72)	74 (67.27)	
IV	52 (14.86)	29 (55.77)	23 (44.23)		22 (42.31)	30 (57.69)	

Abbreviations: TNM stage is based on AJCC. Cases with undefined pM status (MX, *n* = 55) were excluded from the statistical analysis. Significant values are in bold.

**Table 3:** Univariable and multivariable analyses of prognostic factors using the Cox regression model in our cohort.

Variable	Univariable Cox				Multivariable Cox			
	HR	CI Lower 95%	CI Upper 95%	*p*-Value	HR	CI Lower 95%	CI Upper 95%	*p*-Value
IL-6	1.935	1.038	3.611	0.038	1.748	0.906	3.373	0.096
RBP4	4.583	2.445	8.592	<0.0001	3.398	1.775	6.505	<0.0001
Age	1.046	1.015	1.078	0.004	1.034	1.0	1.068	0.048
Gender	2.578	1.311	5.071	0.006	1.922	0.956	3.864	0.067
TNM	3.447	1.938	6.131	<0.0001	3.025	1.66	5.513	<0.0001
Obesity	1.13	0.612	2.087	0.696	-	-	-	-
pN status	3.295	1.852	5.86	<0.0001	-	-	-	-

**Table 4:** Univariable and multivariable analyses of prognostic factors using the Cox regression model in the TCGA cohort.

Variable	Univariable Cox				Multivariable Cox			
	HR	CI Lower 95%	CI Upper 95%	*p*-Value	HR	CI Lower 95%	CI Upper 95%	*p*-Value
IL-6	0.901	0.815	0.997	0.044	-	-	-	-
RBP4	0.982	0.871	1.108	0.773	-	-	-	-
Age	1.03	1.011	1.05	0.002	1.04	1.02	1.06	<0.0001
Gender	1.284	0.816	2.022	0.28	-	-	-	-
TNM	2.519	1.579	4.017	<0.0001	3.116	1.931	5.027	<0.0001
pN status	2.339	1.477	3.704	<0.0001	-	-	-	-

**Table 5:** Top 50 genes showing a positive correlation with *IL-6* identified using Spearman’s rank correlation analysis.

*IL-6* (+) Correlated Gene	Cytoband	Spearman’s Correlation	*p* Value	*IL-6* (+) Correlated Gene	Cytoband	Spearman’s Correlation	*p* Value
*IL11*	19q13.42	0.765	5.83 × 10^−102^	*GPR84*	12q13.13	0.608	2.37 × 10^−54^
*SOCS3*	17q25.3	0.753	9.05 × 10^−97^	*FCAR*	19q13.42	0.603	3.64 × 10^−53^
*IL24*	1q32.1	0.742	1.34 × 10^−92^	*CCL8*	17q12	0.601	7.69 × 10^−53^
*CXCL8*	4q13.3	0.738	3.66 × 10^−91^	*PAPPA*	9q33.1	0.601	9.60 × 10^−53^
*CSF2*	5q31.1	0.724	2.24 × 10^−86^	*ADAMTS4*	1q23.3	0.600	1.48 × 10^−52^
*OSM*	22q12.2	0.721	3.34 × 10^−85^	*SELE*	1q24.2	0.598	4.18 × 10^−52^
*PTGS2*	1q31.1	0.710	1.07 × 10^−81^	*NR4A3*	9q31.1	0.596	8.60 × 10^−52^
*MMP3*	11q22.2	0.708	6.63 × 10^−81^	*HSD11B1*	1q32.2	0.595	1.37 × 10^−51^
*CSF3*	17q21.1	0.706	3.81 × 10^−80^	*FPR1*	19q13.41	0.595	1.85 × 10^−51^
*CXCL5*	4q13.3	0.685	6.51 × 10^−74^	*PROK2*	3p13	0.589	3.74 × 10^−50^
*MMP1*	11q22.2	0.682	8.15 × 10^−73^	*LILRA5*	19q13.42	0.585	2.35 × 10^−49^
*IL1B*	2q14.1	0.673	2.25 × 10^−70^	*CCL4*	17q12	0.585	2.43 × 10^−49^
*CCL2*	17q12	0.655	1.42 × 10^−65^	*SERPINE1*	7q22.1	0.583	4.88 × 10^−49^
*CCL3*	17q12	0.650	3.82 × 10^−64^	*PRR16*	5q23.1	0.580	2.21 × 10^−48^
*PLAU*	10q22.2	0.644	1.07 × 10^−62^	*PDPN*	1p36.21	0.578	4.03 × 10^−48^
*HCAR2*	12q24.31	0.641	4.90 × 10^−62^	*CCL3L1*	17q12	0.578	4.27 × 10^−48^
*FPR2*	19q13.41	0.626	2.02 × 10^−58^	*TFPI2*	7q21.3	0.576	1.21 × 10^−47^
*BCL2A1*	15q25.1	0.620	4.85 × 10^−57^	*TREM1*	6p21.1	0.574	3.02 × 10^−47^
*TNFAIP6*	2q23.3	0.620	5.66 × 10^−57^	*CCN4*	8q24.22	0.571	1.07 × 10^−46^
*STC1*	8p21.2	0.619	7.72 × 10^−57^	*AQP9*	15q21.3	0.566	9.93 × 10^−46^
*PI15*	8q21.13	0.616	4.78 × 10^−56^	*KCNJ15*	21q22.13-q22.2	0.564	2.75 × 10^−45^
*S100A8*	1q21.3	0.614	1.15 × 10^−55^	*CYTL1*	4p16.2	0.562	7.00 × 10^−45^
*CXCL6*	4q13.3	0.614	1.51 × 10^−55^	*FCGR2A*	1q23.3	0.554	1.87 × 10^−43^
*HCAR3*	12q24.31	0.610	8.46 × 10^−55^	*CSF3R*	1p34.3	0.554	2.02 × 10^−43^
*SLC2A3*	12p13.31	0.610	1.20 × 10^−54^	*MEDAG*	13q12.3	0.552	3.54 × 10^−43^

**Table 6:** Top 50 genes showing a negative correlation with *IL-6* identified using Spearman’s rank correlation analysis.

*IL-6* (−) Correlated Gene	Cytoband	Spearman’s Correlation	*p* Value	*IL-6* (−) Correlated Gene	Cytoband	Spearman’s Correlation	*p* Value
*HNF1B*	17q12	−0.356	4.10 × 10^−17^	*NGEF*	2q37.1	−0.292	9.47 × 10^−12^
*IL17RE*	3p25.3	−0.356	4.39 × 10^−17^	*ARHGAP8*	22q13.31	−0.292	9.73 × 10^−12^
*WNK4*	17q21.2	−0.350	1.49 × 10^−16^	*RAVER2*	1p31.3	−0.291	1.04 × 10^−11^
*C14ORF93*	14q11.2	−0.328	1.36 × 10^−14^	*ILDR1*	3q13.33	−0.291	1.06 × 10^−11^
*FOXD2-AS1*	1p33	−0.326	1.88 × 10^−14^	*SIRT5*	6p23	−0.291	1.07 × 10^−11^
*PPFIBP2*	11p15.4	−0.323	3.67 × 10^−14^	*PGAP3*	17q12	−0.289	1.44 × 10^−11^
*IHH*	2q35	−0.322	4.12 × 10^−14^	*HSD11B2*	16q22.1	−0.287	2.00 × 10^−11^
*CEBPA-DT*	19q13.11	−0.314	1.92 × 10^−13^	*CRACDL*	2q11.2	−0.287	2.15 × 10^−11^
*NR1I2*	3q13.33	−0.313	2.08 × 10^−13^	*PRKCZ*	1p36.33	−0.287	2.31 × 10^−11^
*TACC2*	10q26.13	−0.310	3.63 × 10^−13^	*CC2D1A*	19p13.12	−0.286	2.42 × 10^−11^
*ZNF768*	16p11.2	−0.310	4.03 × 10^−13^	*SHD*	19p13.3	−0.286	2.63 × 10^−11^
*FGFR3*	4p16.3	−0.308	6.06 × 10^−13^	*HNF1A-AS1*	12q24.31	−0.284	3.72 × 10^−11^
*BDH1*	3q29	−0.307	6.83 × 10^−13^	*DAPK2*	15q22.31	−0.282	5.03 × 10^−11^
*EVPL*	17q25.1	−0.306	7.81 × 10^−13^	*COQ8A*	1q42.13	−0.280	6.64 × 10^−11^
*AKAP1*	17q22	−0.304	1.08 × 10^−12^	*CCHCR1*	6p21.33	−0.280	6.80 × 10^−11^
*MKRN1*	7q34	−0.304	1.18 × 10^−12^	*ANKS4B*	16p12.2	−0.280	7.05 × 10^−11^
*PPP1R1B*	17q12	−0.303	1.28 × 10^−12^	*TMEM184A*	7p22.3	−0.279	7.41 × 10^−11^
*ZNF69*	19p13.2	−0.302	1.76 × 10^−12^	*ARHGEF5*	7q35	−0.278	8.93 × 10^−11^
*ERBB3*	12q13.2	−0.300	2.26 × 10^−12^	*NDUFA10*	2q37.3	−0.278	9.17 × 10^−11^
*CCDC183*	9q34.3	−0.297	3.63 × 10^−12^	*EIF4EBP3*	5q31.3	−0.278	9.23 × 10^−11^
*SGK2*	20q13.12	−0.294	6.30 × 10^−12^	*ACVR1B*	12q13.13	−0.277	1.06 × 10^−10^
*AP1M2*	19p13.2	−0.294	6.53 × 10^−12^	*TRABD2A*	2p11.2	−0.276	1.30 × 10^−10^
*PLEKHA6*	1q32.1	−0.292	8.76 × 10^−12^	*USH1C*	11p15.1	−0.275	1.49 × 10^−10^
*MYO7B*	2q14.3	−0.292	9.00 × 10^−12^	*ERBB2*	17q12	−0.275	1.54 × 10^−10^
*EPB41L4B*	9q31.3	−0.292	9.03 × 10^−12^	*ZSWIM5*	1p34.1	−0.274	1.88 × 10^−10^

**Table 7:** Top 50 genes positively associated with RBP4 based on Spearman’s rank correlation analysis.

RBP4 (+) Correlated Gene	Cytoband	Spearman’s Correlation	*p* Value	RBP4 (+) Correlated Gene	Cytoband	Spearman’s Correlation	*p* Value
*COLEC11*	2p25.3	0.369	2.19 × 10^−18^	*OTUD5*	Xp11.23	0.319	7.83 × 10^−14^
*CDIPT*	16p11.2	0.368	2.95 × 10^−18^	*LSAMP*	3q13.31	0.319	8.05 × 10^−14^
*DDAH2*	6p21.33	0.367	3.61 × 10^−18^	*ZNF853*	7p22.1	0.318	8.74 × 10^−14^
*FZD8*	10p11.21	0.360	1.61 × 10^−17^	*RNF32-DT*	7q36.3	0.317	1.06 × 10^−13^
*RAMP2*	17q21.2	0.358	2.61 × 10^−17^	*GLI1*	12q13.3	0.317	1.08 × 10^−13^
*ITGB5*	3q21.2	0.355	5.04 × 10^−17^	*FBLN1*	22q13.31	0.316	1.24 × 10^−13^
*SLC29A3*	10q22.1	0.353	8.12 × 10^−17^	*NFASC*	1q32.1	0.316	1.30 × 10^−13^
*TGFB1I1*	16p11.2	0.350	1.67 × 10^−16^	*TFEB*	6p21.1	0.315	1.54 × 10^−13^
*TBC1D25*	Xp11.23	0.346	3.57 × 10^−16^	*GFRA3*	5q31.2	0.314	1.78 × 10^−13^
*APBB1*	11p15.4	0.346	3.59 × 10^−16^	*USP11*	Xp11.3	0.314	1.81 × 10^−13^
*TCF7L1*	2p11.2	0.342	8.46 × 10^−16^	*TCEAL3*	Xq22.2	0.314	1.83 × 10^−13^
*ELN*	7q11.23	0.339	1.45 × 10^−15^	*PDLIM4*	5q31.1	0.314	1.95 × 10^−13^
*MED29*	19q13.2	0.339	1.50 × 10^−15^	*NACAD*	7p13	0.312	2.83 × 10^−13^
*SALL4*	20q13.2	0.333	5.08 × 10^−15^	*PODN*	1p32.3	0.312	2.89 × 10^−13^
*CST3*	20p11.21	0.333	5.10 × 10^−15^	*PLAT*	8p11.21	0.311	3.00 × 10^−13^
*TSPY26P*	20q11.21	0.327	1.67 × 10^−14^	*USP27X*	Xp11.23	0.311	3.23 × 10^−13^
*HHIP*	4q31.21	0.326	2.08 × 10^−14^	*APBA1*	9q21.12	0.310	3.87 × 10^−13^
*RBFOX1*	16p13.3	0.326	2.09 × 10^−14^	*NMUR1*	2q37.1	0.309	4.76 × 10^−13^
*LRRC32*	11q13.5	0.325	2.38 × 10^−14^	*RUSC2*	9p13.3	0.308	5.70 × 10^−13^
*INMT*	7p14.3	0.323	3.18 × 10^−14^	*TAFA5*	22q13.32	0.308	5.79 × 10^−13^
*CPZ*	4p16.1	0.323	3.55 × 10^−14^	*SLC24A3*	20p11.23	0.307	7.31 × 10^−13^
*PTCH2*	1p34.1	0.322	3.98 × 10^−14^	*BMERB1*	16p13.11	0.306	7.47 × 10^−13^
*FRZB*	2q32.1	0.322	4.22 × 10^−14^	*KIAA1755*	20q11.23	0.306	8.41 × 10^−13^
*TEAD3*	6p21.31	0.319	6.87 × 10^−14^	*SCUBE3*	6p21.3	0.306	8.70 × 10^−13^
*TFE3*	Xp11.23	0.319	7.78 × 10^−14^	*ACTB*	7p22.1	0.305	8.97 × 10^−13^

**Table 8:** Top 50 genes negatively associated with *RBP4* based on Spearman’s rank correlation analysis.

*RBP4* (−) Correlated Gene	Cytoband	Spearman’s Correlation	*p* Value	*RBP4* (−) Correlated Gene	Cytoband	Spearman’s Correlation	*p* Value
*HSPA4L*	4q28.1	−0.398	2.71 × 10^−21^	*MAP2K1*	15q22.31	−0.294	6.63 × 10^−12^
*PPAT*	4q12	−0.365	6.35 × 10^−18^	*INTS13*	12p11.23	−0.294	6.65 × 10^−12^
*GUF1*	4p12	−0.346	3.50 × 10^−16^	*PDS5A*	4p14	−0.293	7.66 × 10^−12^
*LIAS*	4p14	−0.336	2.47 × 10^−15^	*CCT8*	21q21.3	−0.293	7.69 × 10^−12^
*RPRD1A*	18q12.2	−0.325	2.40 × 10^−14^	*UBE3A*	15q11.2	−0.290	1.33 × 10^−11^
*CBR4*	4q32.3	−0.322	4.06 × 10^−14^	*TIMM21*	18q22.3	−0.289	1.46 × 10^−11^
*POLR3G*	5q14.3	−0.321	5.49 × 10^−14^	*TM7SF3*	12p11.23	−0.289	1.58 × 10^−11^
*TMEM33*	4p13	−0.320	5.83 × 10^−14^	*MRPS27*	5q13.2	−0.289	1.59 × 10^−11^
*SFXN1*	5q35.2	−0.316	1.21 × 10^−13^	*CDC7*	1p22.1	−0.287	2.17 × 10^−11^
*ME2*	18q21.2	−0.316	1.38 × 10^−13^	*EXOSC9*	4q27	−0.286	2.38 × 10^−11^
*OIP5-AS1*	15q15.1	−0.315	1.46 × 10^−13^	*MDH1*	2p15	−0.286	2.46 × 10^−11^
*PAICS*	4q12	−0.312	2.77 × 10^−13^	*TIPIN*	15q22.31	−0.286	2.59 × 10^−11^
*MREG*	2q35	−0.311	3.18 × 10^−13^	*DNAJC16*	1p36.21	−0.285	2.86 × 10^−11^
*C12ORF60*	12p12.3	−0.310	3.75 × 10^−13^	*PMS1*	2q32.2	−0.285	3.10 × 10^−11^
*SLC35F2*	11q22.3	−0.307	7.12 × 10^−13^	*LARS1*	5q32	−0.283	3.89 × 10^−11^
*CIAO2A*	15q22.31	−0.306	8.36 × 10^−13^	*COPS2*	15q21.1	−0.283	4.23 × 10^−11^
*GRSF1*	4q13.3	−0.305	9.43 × 10^−13^	*VRK2*	2p16.1	−0.282	4.68 × 10^−11^
*CENPE*	4q24	−0.303	1.35 × 10^−12^	*MTIF2*	2p16.1	−0.282	4.69 × 10^−11^
*UBA6*	4q13.2	−0.302	1.54 × 10^−12^	*CDC27*	17q21.32	−0.282	4.70 × 10^−11^
*FASTKD1*	2q31.1	−0.302	1.75 × 10^−12^	*NCAPG*	4p15.31	−0.282	4.90 × 10^−11^
*TFRC*	3q29	−0.301	1.86 × 10^−12^	*RAD54B*	8q22.1	−0.282	5.16 × 10^−11^
*ETF1*	5q31.2	−0.296	4.50 × 10^−12^	*TMEM161B*	5q14.3	−0.281	5.46 × 10^−11^
*SKA1*	18q21.1	−0.296	4.70 × 10^−12^	*SELENOI*	2p23.3	−0.281	5.91 × 10^−11^
*CHAF1B*	21q22.12-q22.13	−0.295	5.30 × 10^−12^	*SRRM1*	1p36.11	−0.280	6.30 × 10^−11^
*NARS1*	18q21.31	−0.295	5.83 × 10^−12^	*TMX2*	11q12.1	−0.279	7.61 × 10^−11^

## Data Availability

The datasets generated during and/or analyzed during the current study are available from the corresponding author on reasonable request.
